# Complex regional pain syndrome: a recent update

**DOI:** 10.1186/s41038-016-0066-4

**Published:** 2017-01-19

**Authors:** En Lin Goh, Swathikan Chidambaram, Daqing Ma

**Affiliations:** Anaesthetics, Pain Medicine and Intensive Care, Department of Surgery and Cancer, Faculty of Medicine, Imperial College London, Chelsea and Westminster Hospital, 369 Fulham Road, London, SW10 9NH UK

**Keywords:** CRPS, Epidemiology, Pathophysiology, Treatment, Future ﻿therapy﻿

## Abstract

Complex regional pain syndrome (CRPS) is a debilitating condition affecting the limbs that can be induced by surgery or trauma. This condition can complicate recovery and impair one’s functional and psychological well-being. The wide variety of terminology loosely used to describe CRPS in the past has led to misdiagnosis of this condition, resulting in poor evidence-base regarding the treatment modalities available and their impact. The aim of this review is to report on the recent progress in the understanding of the epidemiology, pathophysiology and treatment of CRPS and to discuss novel approaches in treating this condition.

## Background

Complex regional pain syndrome (CRPS) is a chronic neurological condition involving the limbs that is characterised by severe pain along with sensory, autonomic, motor and trophic impairment [1, 2]. This condition may be induced by surgery, trauma or minor injury and has a varying course, ranging from mild and self-limiting, to chronic disease, which impairs activities of daily living and health-related quality of life. The occurrence of CRPS following elective or emergency extremity surgery may complicate recovery and post-operative management. This increases the probability of a poorer outcome and exerts a large financial burden on the healthcare system. Due to the complexity and broad spectrum of symptoms, patients with CRPS require input from various clinical specialties including orthopaedic surgeons, anaesthetists, rheumatologists and rehabilitation physicians. This mini-review aims to provide an update on the recent progress in the understanding of the epidemiology, pathophysiology and treatment of CRPS and to discuss novel approaches in treating this condition.

## Review

### Diagnostic criteria

CRPS is a clinical diagnosis made based on the findings during the history and physical examination of the patient, for which diagnostic criteria including the Orlando Criteria for Complex Regional Pain Syndrome and The Budapest Clinical Diagnostic Criteria for Complex Regional Pain Syndrome by the International Association for the Study of Pain (IASP) have been developed [1]. CRPS can be classified into two types: CRPS types I and II that are characterised by the absence or presence of identifiable nerve injury. CRPS type I is a syndrome that usually develops after an initiating noxious event, is not limited to the distribution of a single peripheral nerve, and is disproportionate to the inciting event. It is associated with oedema, changes in skin blood flow, abnormal sudomotor activity in the region of the pain, allodynia and hyperalgesia and commonly involves the distal aspect of the affected extremity or with a distal to proximal gradient. CRPS type II can be defined as a burning pain, allodynia and hyperpathia occurring in a region of the limb after partial injury of a nerve or one of its major branches innervating that region [1, 2].

### Epidemiology

#### Overview

Although the diagnostic criteria for CRPS were put forward in 1994, limited data from epidemiological studies are available before 2000. Sandroni et al. conducted the first population-based study of CRPS in 2003, where they reviewed and validated potential cases of CRPS of the local population of Olmsted County over a 10-year period using the IASP and Harden criteria [3]. The incidence rate of CRPS type I was 5.46 per 100000 person-years, and the incidence rate of CRPS type II was 0.82 per 100000 person-years, giving rise to a combined incidence rate for both CRPS types I and II of 6.28 per 100000 person-years. However, a subsequent population-based study by de Mos et al. estimated the combined incidence rate of CRPS to be approximately four times greater at 26.2 per 100000 person-years [4]. This has been attributed to differences in ethnic and socio-economic background of the cohort as well as the application of the diagnostic criteria. In contrast to Sandroni et al., the study by de Mos et al. did not require all cases to fulfil the diagnostic criteria but instead retained cases based on confirmation of the diagnosis by the general practitioner or specialist. Furthermore, the retrospective application of the IASP criteria to information on electronic charts as performed by Sandroni et al. may have been overly strict. CRPS occurs most frequently in individuals aged between 61 and 70 years and demonstrates a female predilection, affecting three times more females than males [4]. There appears to be an increased preponderance for the upper limbs with a ratio of 3:2 compared to the lower limbs. Risk factors for this condition include menopause, individuals with a history of migraine, osteoporosis, asthma and angiotensin-converting enzyme (ACE) inhibitor therapy and individuals with an elevated intracast pressure due to a tight case or extreme positions [5–7]. Furthermore, the prognosis of CRPS is poorer in smokers compared to non-smokers [8].

#### Surgery

The development of CRPS following surgery is a major cause of concern as this complicates post-operative management and has significant clinical ramifications. As such, rapid diagnosis and treatment are required to prevent the sequelae such as swelling, atrophy, osteoporosis, pseudo-arthrosis, joint stiffness and tendon adhesions. Operative procedures of the shoulder, distal radius, carpal tunnel and Dupuytren’s contracture have been shown to be associated with the manifestation of CRPS. The incidence of CRPS following shoulder, distal radius, carpal tunnel and Dupuytren’s contracture surgery is estimated to be between 0.9 and 11%, 22 to 39%, 2 to 5% and 4.5 to 40%, respectively (Table 1) [9–18]. Although less studied, surgical treatment of the lower limb is also associated with the development of CRPS. In a prospective study of patients with tibial fractures, the incidence of CRPS following surgical repair was documented at 31%; 33.3% of patients treated with intramedullary nailing, 28.6% of patients treated with nails and screws and 28.6% of patients treated with external fixation [19]. A recent retrospective study by Rewhorn et al. evaluating the occurrence of CRPS following elective ankle and foot surgery in 390 patients found the overall incidence to be 4.4%; 3.6% for CRPS type I and 1.8% for CRPS type II [18]. Studies investigating the incidence of CRPS following orthopaedic surgery have been limited by the size of the cohort, thus, making it difficult to draw a reliable estimate regarding the true prevalence of this syndrome. Furthermore, findings from the majority of these studies are highly susceptible to a type I error due to the lack of a gold standard diagnostic criteria in diagnosing this condition.

**Table 1:** Reported incidence of CRPS following surgical procedures of the upper and lower limb

Region	Operation	Study	Incidence
Upper limb	Shoulder	Chalmers et al. 2014 [9]	11.1% (1:8)
		Arndt et al. 2012 [10]	3.0% (3:97)
		Gonzalez et al. 2011 [11]	0.9% (35:3975)
		Bishop et al. 2005 [127]	1.3% (1:79)
		Borgeat et al. 2001 [12]	1.0% (5:516)
	Carpal tunnel release	Shinya et al. 1995 [13]	1.9 (2:105)
		Litchman et al. 1979 [14]	5.0% (5:95)
		MacDonald et al. 1978 [15]	2.2% (4:182)
	Dupuytren’s contracture	Lily and Stern 2010 [16]	2.0% (1:49)
		Bulstrode et al. 2005 [17]	2.4% (6:247)
Lower limb	Tibial	Sarangi et al. 1993 [19]	31% (9:20)
	Ankle and foot	Rewhorn et al. 2014 [18]	4.4% (17:373)

#### Fracture

Fractures appear to be a common inciting event for the development of CRPS. A recent study by Beerthuizen et al. investigated the occurrence of CRPS type I after 1 year in 596 patients who had suffered fractures of the upper or lower extremity [20]. In this study, the overall incidence of CRPS type I was 7.0, with 15.2% of cases occurring after ankle fracture, 2.9% following fifth metatarsal fracture and 7.9% after wrist fracture. No cases of CRPS were described following scaphoid fractures in this study. It is apparent however that the incidence of CRPS varies widely between studies. For instance, the development of CRPS following fractures of the distal radius is reported to range between 1 and 37% (Table 2) [19–24]. In contrast to fractures of the upper extremity, there is limited evidence regarding the incidence of CRPS following fractures of the lower extremity. The only other study to date was conducted by Sarangi et al., who reported an incidence of 30% in their cohort of 30 patients with tibial fractures treated with plaster casts [19]. In most of these patients, the symptoms resolved within 6 months. Despite a substantial amount of data available regarding the incidence of CRPS, a wide variability in findings persists. This can be attributed to inconsistencies in diagnostic criteria used. For instance, Beerthuizen et al. reported marked differences in the number of patients diagnosed with the IASP, Veldmen and Harden and Bruehl criteria, all of which were utilised prior to the introduction of the Budapest criteria for CRPS [20]. As such, it is likely that the differences in sensitivities of these criteria are likely to result in varying rates of misdiagnosis. Moreover, data available from these studies have been limited solely to CRPS type I, and as such, more work is needed to elucidate the incidence of CRPS type II.

**Table 2:** Reported incidence of CRPS following fractures of the upper and lower limbs

Region	Antecedent Event	Study	Incidence
Upper limb	Distal radius fracture	Jellad et al. 2014 [21]	32.2% (29:61)
		Dijkstra et al. 2003 [22]	1.1% (1:87)
	Colles’ fracture	Bickerstaff and Kanis 1994 [23]	28.1% (77:197)
		Atkins et al. 1990 [24]	36.7% (22:38)
	Wrist fracture	Beerthuizen et al. 2012 [20]	7.9% (18:209)
	Scaphoid fracture	Beerthuizen et al. 2012 [20]	0% (0:27)
Lower limb	Tibial fracture	Sarangi et al. 1993 [19]	30% (9:21)
	Ankle fracture	Beerthuizen et al. 2012 [20]	15.2% (21:117)
	Fifth metatarsal fracture	Beerthuizen et al. 2012 [20]	2.9% (3:100)

### Pathophysiology

#### Inflammation

The clinical presentation of the acute phase of CRPS supports the hypothesis that the development of this condition is due to an exaggerated inflammatory response to trauma. Clinical findings of the CRPS-affected limb reveal pain, oedema, erythema, increased temperature and impaired function—the five cardinal signs of inflammation [25]. Tissue trauma triggers the release of pro-inflammatory cytokines such as interleukin(IL)-1β, IL-2, IL-6 and tumour necrosis factor-α (TNF-α) along with neuropeptides including calcitonin gene-related peptide, bradykinin and substance P. These substances increase plasma extravasation and vasodilation, producing the characteristic features of acute CRPS [26, 27].

#### Altered cutaneous innervation

Initial neuronal injury, however imperceptible has been implicated as an important trigger in the development of both CRPS types I and II [28]. This has been supported by studies demonstrating a reduction in C-type and Aδ-type cutaneous afferent neuron fibre density in the CRPS-affected limb compared to the unaffected limb, with these changes primarily affecting nociceptive fibres [29, 30]. The decrease in C-type and Aδ-type fibres was associated with an increase in aberrant fibres of unknown origin, and it has been postulated that the exaggerated pain sensation may be due to altered function of these fibres [30]. One animal study on rats has shown a causal relationship between this neuronal trigger and a reduction in neuron fibre density, highlighting the possibility that altered cutaneous innervation of the CRPS-affected limbs may be a result of an initial neuronal injury [31]. Human studies, however, have been unable to replicate this causative effect, thus, bringing into question whether the reduction in neuron fibre density is an epiphenomenon rather than being directly related to the condition.

#### Central and peripheral sensitisation

Following tissue damage and/or neuronal injury, alterations in the central and peripheral nervous systems lead to increased inflammation, and an enhanced responsiveness to pain. These adaptations act as protective mechanisms to promote avoidance of activities that cause further injury. Within the central nervous system (CNS), persistent and intense noxious stimulation of peripheral nociceptive neurons results in central sensitisation. Accordingly, there is alteration in nociceptive processing in the CNS and increased excitability of secondary central nociceptive neurons in the spinal cord. This is mediated by the release of neuropeptides such as substance P, bradykinin and glutamate by peripheral nerves, which sensitise and increase the activity of local peripheral and secondary central nociceptive neurons resulting in increased pain from noxious stimuli (hyperalgesia) and pain in response to non-noxious stimuli (allodynia) [26, 32, 33]. Research has shown that CRPS patients have a significantly greater windup to repeated stimulation of the affected limb compared to the contralateral limb or other limbs [34, 35].

#### Altered sympathetic nervous system function

In the chronic (cold) phase of the clinical course of CRPS, the CRPS-affected limb is cyanosed and clammy as a result of vasoconstriction and sweating. This suggests that excessive sympathetic nervous system outflow is a driving factor in progression of the condition and maintenance of the pain [36]. Animal studies have observed adrenergic receptor expression on nociceptive fibres following nerve trauma, which may provide a possible mechanism of the sympathetically induced pain. In addition, expression of adrenergic receptors on nociceptive fibres following injury may contribute to sympatho-afferent coupling increasing the pain intensity [28]. This has been demonstrated in patients with sympathetically mediated CRPS pain where high sympathetic nervous system activity increased spontaneous pain by 22% and increased the spatial extent of dynamic and punctate hyperalgesia by 42 and 27% respectively [37].

#### Circulating catecholamines

Variation in the clinical features of CRPS as the condition progresses from the acute (warm) phase to the chronic phase may be attributed to alterations in catecholaminergic mechanisms [28]. During the acute phase, the CRPS-affected limb demonstrates a reduction in the levels of circulating plasma norepinephrine compared to the unaffected limb [38]. As a result, there is compensatory upregulation of peripheral adrenergic receptors causing supersensitivity to circulating catecholamines [39]. Consequently, excessive vasoconstriction and sweating occurs following exposure to catecholamines, giving rise to the characteristic cold and blue extremity seen during the chronic phase.

#### Autoimmunity

The presence of immunoglobulin G (IgG) autoantibodies against surface antigens on autonomic neurons in the serum of patients with CRPS suggests that autoimmunity may play a role in the development of this condition [40, 41]. This is supported by the results of a small pilot trial where patients with CRPS who were given intravenous immunoglobulin treatment demonstrated a significant reduction in pain symptoms when compared with those given a placebo [42].

#### Brain plasticity

Neuroimaging studies of patients with CRPS have demonstrated a decrease in area representing the CRPS-affected limb in the somatosensory cortex compared to the unaffected limb [43, 44]. The sensory representation of the affected limb, as part of the Penfield homunculus is distorted, with shrinkage and shifting of the area [43]. The extent of reorganisation bears significant correlation with the pain intensity and degree of hyperalgesia experienced by the patient, and these alterations return to normal following successful CRPS treatment [43, 45, 46].

#### Genetic factors

Although there is a lack of consensus regarding the influence of genetic factors in CRPS, family studies have suggested a genetic preponderance towards developing this condition. Siblings of CRPS patients under 50 years were at three times higher risk of developing the condition, with a mitochondrial inheritance pattern [47, 48]. Furthermore, the genes of the major histocompatibility complex encoding the human leukocyte antigen (HLA) molecules, HLA-B62 and HLA-DQ8 alleles were found to strongly correlate with the development of CRPS [49].

#### Psychological factors

Due to the prevalence of anxiety and depression in patients with CRPS and the unusual nature of symptoms, psychological factors have been hypothesised to play a role in the development or propagation of CRPS. Puchalski et al. observed a higher occurrence of CRPS following fractures of the distal radius in elderly patients with psychological and/or psychiatric illness, thereby implicating the role of psychological factors [50]. However, evidence regarding this remains inconclusive as other studies have failed to confirm this association, and a definitive causation has yet to be identified [28].

### Management

#### Physical and occupational therapy

Physical and occupational therapy is a key component of the rehabilitation process in patients with CRPS and is recommended as the first-line treatment. Patients can develop kinesophobia and the aim of therapy is to overcome this fear of pain and enable the patient to gain the best functional use of the limb. This program is tailored specifically to each individual and can involve multiple modalities. These include elevation, massage, contrast baths, transcutaneous electrical nerve stimulation, gentle range of motion, isometric strengthening exercise and stress loading of the affected limb along with provision of adequate analgesia. Occupational therapy encourages use of the affected limb in activities of daily living. The use of specialised garments or wrappings may reduce oedema and sensory overload of the affected limb. Mirror box therapy has been shown to reduce neuropathic pain and improve two-point sensation in the affected limb [51].

#### Psychological therapy

Chronic pain affects the health-related quality of life and places a huge emotional and psychological burden on patients. Thus, it is essential for newly diagnosed patients with CRPS to have a discussion with a psychological care provider regarding their condition and its progression as well as the need for active self-management and participation in a care plan. This can be followed up with cognitive behavioural therapy, learning relaxation skills and biofeedback to facilitate rehabilitation, reduce pain intensity and provide patients with more control. It is important to assess and treat patients for concomitant axis I disorders such as major depression, generalised anxiety disorder and post-traumatic stress disorder, which may complicate the rehabilitation process.

#### Medical management

Corticosteroids and non-steroidal anti-inflammatory drugs (NSAIDs) reduce inflammation and have been used in the treatment of CRPS. Randomised controlled trials (RCTs) and case series have reported significant improvements in pain and range of motion in the affected limb following treatment with both oral and intramuscular corticosteroid regimens [52–56]. However, it is evident that these improvements are not experienced by all patients suffering from CRPS, which may be attributed to the multifactorial and heterogeneous nature of the condition [57]. There is currently no evidence of clinically positive effects following treatment with NSAIDs.

The use of anti-oxidants in the treatment of CRPS has been based on the perception that oxygen free radicals generated by the inflammatory process may be a key component of the propagation of the disease process. Topical preparations of anti-oxidants such as dimethyl sulfoxide (DMSO) and *N*-acetylcysteine have shown success in providing pain relief [58, 59]. The significant preventative effect of vitamin C in halting the transition to CRPS can be attributed to its anti-oxidant properties [60, 61]. Vitamin C is currently established as the most efficacious preventative therapy for the development of CRPS and is commonly used perioperatively following extremity surgery [62, 63].

Anti-convulsant drugs such as gabapentin have demonstrated evidence of effectiveness in providing pain relief in acute and chronic neuropathic are commonly used as part of the pharmacological management of CRPS [64, 65]. Studies investigating the efficacy of gabapentin in CRPS type I have reported marked improvements in pain reduction and long-term sensory deficits, thereby supporting the utility of this form of therapy [66, 67]. Although gabapentin has a good safety profile, it is important to be aware of the rare but severe side effects including mood disorders and suicide ideation [68]. Although there is no evidence supporting the long-term effectiveness of anti-convulsants for CRPS, these agents may be useful in providing pain relief in the earlier stage of the disease.

The upregulation of inflammatory pathways in CRPS sensitises excitatory nociceptive pathways that use *N*-methyl-D-aspartic acid (NMDA) as a neurotransmitter [69]. The central sensitisation and alteration of brain plasticity that occurs could potentially be reversed with the use of the NMDA receptor antagonist ketamine, which can be administered topically or intravenously. Placebo-controlled studies have shown both topical and intravenous administration of ketamine to be effective at alleviating pain and inducing complete remission in treatment resistant patients, thereby highlighting the potential of this approach [70–73]. However, side effects including feelings of inebriation, nausea, vomiting, headaches and psychomimetic effects are highly prevalent, and these have hindered the application of ketamine in treating CRPS [74].

Sympathetically mediated pain in CRPS has led to the studies investigating the role adrenergic receptor antagonists or alpha-2 adrenergic agonists in treating this condition. Phenoxybenzamine has shown success in providing complete pain remission, with increased effectiveness when administered in the acute stage, thus emphasising the importance of early recognition of CRPS [75, 76]. There have also been case reports of the efficacy of this approach in patients in whom alternative therapies have failed, although these have been limited to small patient numbers [77]. Alternatively, clonidine, which is an alpha-2 adrenergic agonist, has been reported to relieve localised hyperalgesia and provide extensive analgesia in patients with sympathetically mediated pain [78].

Several treatment strategies have also been used in managing the chronic stage of CRPS. Calcium-channel blockade with nifedipine has been reported to be effective in managing the vasoconstriction occurring in this phase of CRPS [76, 79]. Additionally, the use of gamma-aminobutyric acid-β (GABA) agonist such as baclofen has also been effective in reducing dystonia and pain while improving functionality and quality of life in patients with chronic CRPS [80–82]. A recent study looking into combined neuromodulation with baclofen as an adjunct to spinal cord stimulation (SCS) therapy demonstrated effectiveness in decreasing pain intensity and dystonia, suggesting the need for further larger scale trials [83].

As CRPS progresses, there is localised bone resorption and remodelling, which can lead to nociceptive bone pain, osteopenia and osteoporosis. In addition, reduction in bone mineral density occurs as a result of lack of use of the affected limb. Calcitonin preserves bone mass, has effects on microvasculature and has anti-nociceptive effects, which have been found to be effective in treating acute and chronic pain [84–86]. Bisphosphonates inhibit osteoclasts, slowing down bone resorption and increasing bone mineral density and are well-established to be effective at providing pain relief [87–91]. A review in 2010 concluded bisphosphonates as the only medications with clear benefits in treating CRPS [92]. One long-term complication of bisphosphonate therapy is the development of pathologic fractures, which is thought to be due to compromise in microstructural properties of bone arising from the accumulation of microcracks [93]. However, CRPS patients only require a few months of treatment and therefore, are at minimal risk. Bisphosphonates are contraindicated in patients with decreased renal function, oesophageal motility disorders, peptic ulcer disease and poor dentition. As there is the risk of jaw osteonecrosis, patients should be told to report any tooth, jaw, face or head discomfort during treatment [68, 94].

There are contrasting views regarding the use of opioid therapy in the treatment of CRPS. While opioid therapy is useful in the acute phase of tissue injury, long-term use for both peripheral and central neuropathic pain is less efficacious and requires larger doses [95]. Although the safety and effectiveness of opioids in treating neuropathic pain have been documented, higher doses and long-term use can result in tolerance, addiction, misuse, immunosuppression, endocrine dysfunction and overdoses leading to death [96].

The discovery of autoantibodies against adrenergic receptors suggesting that CRPS has an autoimmune component provides the basis for the use of intravenous immunoglobulin (IVIG), which is a potent anti-inflammatory and immune-modulator [40, 41]. A RCT of 13 patients with chronic CRPS comparing low-dose IVIG with intravenous normal saline reported pain relief in the 12 patients who completed the trial at 6–19 days following treatment [42].

#### Anaesthesia therapy

An alternative approach studied involves the use of sympathetic blockade, which has diagnostic and therapeutic benefits. Sympathetic blocks aim to alleviate the sympathetically mediated pain and can be used in combination with botulinum toxin to prolong the duration of analgesia. Recent evidence has shown sympathetic blockade to provide substantial pain reduction as well as longer analgesic duration, which enable patients to improve participation in functional therapies [97–99]. However, there remains a lack of definitive evidence regarding the efficacy of sympathetic blockade overall, and this approach has yet to be shown to be curative [100].

#### Surgical management

Neuromodulation may also play a role in treating CRPS, especially in patients in unresponsive to sympathetic blockade. One RCT reported SCS and physiotherapy to be significantly more effective at pain relief compared with physiotherapy on its own at 6 months and 2 years although this effectiveness diminished at long-term follow up of 5 years [101]. However, the prevalence of complications is high, including lead displacement, pulse-generator pocket revision, pulse-generator failure and infection [102, 103]. In the majority of patients, SCS is associated with sustained improvements in functional capability, quality of life, depression and pain levels [103, 104].

Sympathectomy can be performed as an extension of temporary sympathetic blockade in patients who have good but transient relief from sympathetic blockade. This involves severing the sympathetic chains or stellate ganglion using chemicals, radiofrequency or open surgical techniques to prolong analgesia. Chemical sympathectomy is carried out using alcohol or phenol injections to destroy the sympathetic chain but this method has variable outcomes with limited evidence to support its effectiveness [105]. Radiofrequency sympathectomy provides a longer lasting pain relief, with 40% of patients reporting greater than 50% pain reduction after a year [106]. Complications of sympathectomy are common such as post-sympathectomy neuralgia, anhydrosis and Horner’s syndrome. Given the permanent nature of this approach, sympathectomy is generally considered only in patients where alternative treatment options have failed.

Amputation in CRPS may be indicated due to pain, limb dysfunction, gangrene, infection or ulcers [107]. The majority of patients report a reduction in pain along with improvements in mobility and sleep following amputation of the affected limb, but many suffer from phantom pain and recurrence in the residual limb [107, 108].

### Emerging treatments

#### Immunomodulation

Chronic regional and neurogenic inflammation are thought to play a key role in the initiation and propagation of CRPS [28, 109]. Patients suffering from this condition display systemic elevation of pro-inflammatory cytokines and a corresponding reduction in the anti-inflammatory cytokine IL-10 [110]. Anti-cancer drugs such as lenalidomide and thalidomide possess anti-inflammatory and immunomodulatory effects and have shown promise in alleviating this condition. In the open-label study of thalidomide, pain relief was reported in approximately one-third of the participants, which was evident within 4 to 6 weeks of treatment commencing [111]. Similarly, patients receiving lenalidomide reported significant improvements in pain and functional scores within 12 weeks, which persisted for one year [112]. Promisingly, patients accruing the greatest benefit from these agents are those with very high pain scores and treatment-refractory disease. Despite the recent failure of the phase IIb trial of lenalidomide to show any benefit over the placebo, it may be premature to discard this approach [113]. It is increasingly apparent that there are distinct CRPS populations, so subgroups of patients with elevated pro-inflammatory cytokine levels may stand to benefit from a trial of this treatment.

#### Hyperbaric oxygen therapy

The anti-nociceptive effect of hyperbaric oxygen therapy (HBOT) has been well-documented in animal models. This is conferred via neural nitric oxide-dependent release of the endogenous opioid, dynorphin, which subsequently activates k- and μ-opioid receptors [114]. A RCT was designed to evaluate the efficacy of HBOT in treating 71 patients with post-traumatic CRPS of the wrist [115]. In this study, patients receiving 15 daily 90-min HBOT sessions demonstrated substantially lower visual analogue scale (VAS) scores 45 days following treatment. Pain relief occurred rapidly with marked improvements in VAS scores evident by the end of the first day. Moreover, HBOT was shown to be effective at reducing oedema and improving range of motion. However, it is important to note that the generalization of these findings must be treated with caution since treatment was commenced within a month and a half of the initial injury. As such, further work is necessary to replicate the beneficial effects of this approach in patients with more chronic injuries.

#### Botulinum toxin-A (BTX-A)

BTX-A has been shown to confer pain relief in neuropathic pain, which complicates disorders of the central and peripheral nervous system and may therefore demonstrate efficacy in managing CRPS. Kharkar et al. investigated the efficacy of BTX-A in providing pain relief in 37 subjects with CRPS suffering from focal tonic dystonia [116]. In this study, 97% of patients reported significant pain relief with a 43% reduction in the mean pain score after 4 weeks of treatment. Although this was a retrospective study lacking a control group, the results are promising and call for further clinical trials. It is important to note that despite the growing use of BTX-A in clinical practice, there is still limited information to guide the choice, formulation and dose of the toxin, which are mainly based on the experience of the clinician.

#### Plasma exchange

Recent developments in the understanding of the autoimmune aetiology of CRPS have highlighted the potential use of plasma exchange therapy, which has demonstrated benefit in other autoimmune disorders. A retrospective case series of 33 patients with CRPS receiving plasma exchange was conducted by Aradillas et al. to assess this approach [117]. In this case series, 91% of patients reported a significant median pain reduction of 64% following therapy. Additionally, weekly treatment was shown to be successful in maintaining pain relief in 45% of patients. This study advocates large, randomised placebo-controlled trials to validate and expand on the findings.

#### Future therapy

As our understanding of the pathophysiology of CRPS continues to grow, so too does the scope for the finding of new or existing agents to target the different disease mechanisms. The neurogenic inflammation that occurs in CRPS can be attributed in part to the activation of microglia, which is mediated by Toll-like receptor (TLR) signalling. Naltrexone has antagonistic effects at the TLR-4 and hypothetically suppress inflammation. A recent case series of two patients found low-dose naltrexone to be effective in reducing pain with minimal side effects and a RCT is currently ongoing to investigate this further [118, 119].

In neuropathic pain models, activated microglia express cannabinoid receptor-2 (CB-2) and chemokine fractalkine receptor (CX3CR1) [120, 121]. Both receptors play a significant role in microglial activation and neuroinflammation, and it is hypothesised that regulating the signalling of these two receptors with a CB2 agonist could modulate the pain and inflammation. In the rat model of CRPS, the novel CB2 agonist MDA7 was found to suppress peripheral oedema, microglial activation and receptor expression in the spinal cord, thereby corroborating the hypothesis [122]. Human studies are necessary to elucidate the efficacy of this agent in treating CRPS.

NSAIDs and corticosteroids have been used in CRPS with the aim of limiting pain and inflammation. The novel anti-inflammatory agent, polydeoxyribonucleotide is a low molecular weight deoxyribonucleic acid complex that acts as a selective agonist against the adenosine A2A receptor. This leads to a decrease in the secretion of inflammatory cytokines such as TNF-α, macrophage inflammatory protein 1α and IL-6 along with an increase in IL-10 [123]. Furthermore, activation of this receptor induces endothelial cell proliferation and migration to promote tissue regeneration [124, 125]. In a recently published case report, polydeoxyribonucleotide treatment was associated with rapid pain relief, which underlines the potential of this approach [126].

## Conclusion

### Future prospects

Although there has yet to be a successful treatment for CRPS to date, years of research have provided us with many valuable lessons and our understanding of this condition continues to grow. It is evident that a CRPS population is heterogeneous, with distinct subgroups that exhibit different clinical and biochemical features, thereby exhibiting a varying response to treatment. Moreover, the evidence-base regarding CRPS type II remains scarce in contrast to CRPS type I. Hence, future work is needed to elucidate the subgroups of patients who would benefit the most from currently available treatment. Given the complex nature of this syndrome, it is unlikely that targeting a specific mechanism will be effective. As with other chronic disorders, the future of CRPS treatment may lie in combination therapy and studies investigating this will be necessary.

### Summary

The complex pathophysiology of CRPS remains a challenge for clinicians and researchers alike in developing treatments to successfully combat this severe, life-threatening condition. Due to the multifactorial nature of this condition, animal models that can simulate the disease process are lacking, which is further compounded by our limited understanding of the mechanisms involved. This has hindered the development of new therapies, leading clinicians to adopt a trial and error approach towards managing this syndrome. Hence, the majority of studies evaluating novel approaches have been restricted to case series or small pilot studies. The recent declaration by the United States Food and Drug Administration of CRPS as an official disease has given us renewed hope, as this has been a catalyst for new drug development.

## Abbreviations

ACE: Angiotensin-converting enzyme
BTX-A: Botulinum toxin-A
CB-2: Cannabinoid receptor-2
CNS: Central nervous system
CRPS: Complex regional pain syndrome
CX3CR1: Chemokine fractalkine receptor
DMSO: Dimethyl sulfoxide
GABA: Gamma-aminobutyric acid-β
HBOT: Hyperbaric oxygen therapy
HLA: Human leukocyte antigen
IASP: International Association for the Study of Pain
IgG: Immunoglobulin G
IL: InterleukiN
IVIG: Intravenous immunoglobulin
NMDA: *N*-methyl-d-aspartic acid
NSAID: Non-steroidal anti-inflammatory drug
RCT: Randomised controlled trial
SCS: Spinal cord stimulation
TLR: Toll-like receptor
TNF-α: Tumour necrosis factor-α
VAS: Visual analogue scale
